# Evolution of the proinflammatory receptor TREM-1 in mammals reveals signatures of pathogen-driven conflict

**DOI:** 10.64898/2026.08.05.743136

**Published:** 2026-08-10

**Authors:** Omoshola Aleru, Kaitlyn E. Bunn, Matthew F. Barber

## Abstract

Animal immune cells express a range of surface receptors that promote the detection of diverse host and pathogen-derived molecules. The triggering receptors expressed on myeloid cells (TREMs) encompass a family of cell surface receptors involved in the modulation of immune signaling cascades. Mammalian TREM-1 has emerged as a critical mediator of antibacterial immune defense and inflammatory disease, yet much remains unknown regarding its evolution and relevant molecular interactions. Here we applied a comparative phylogenetic approach to investigate patterns of divergence and natural selection among mammalian TREM-1 orthologs. We identify evidence of repeated positive selection acting within the extracellular ligand binding domain of TREM-1 among primates, rodents, and particularly bats. Structural simulations further suggest that genetic variation in TREM-1 impacts recognition of putative host and microbial ligands, with implications for downstream signaling functions. Together our findings identify patterns of rapid divergence in mammalian TREM-1, suggesting a history of evolutionary conflict in response to pathogen antagonism.

## Full Text Availability

The license terms selected by the author(s) for this preprint version do not permit archiving in PMC. The full text is available from the preprint server.

